# Of genes and microbes: solving the intricacies in host genomes

**DOI:** 10.1007/s13238-018-0532-9

**Published:** 2018-04-02

**Authors:** Jun Wang, Liang Chen, Na Zhao, Xizhan Xu, Yakun Xu, Baoli Zhu

**Affiliations:** 1 CAS Key Laboratory of Pathogenic Microbiology and Immunology, Institute of Microbiology, Chinese Academy of Science, Beijing, 100101 China; 2 University of Chinese Academy of Sciences, Beijing, 100049 China; 3 Collaborative Innovation Centre for Diagnosis and Treatment of Infectious Diseases First Affiliated Hospital, School of Medicine, Zhejiang University, Hangzhou, 310058 China

**Keywords:** gut microbiota, host genetics, quantitative genetics, gene-microbiome association

## Abstract

Microbiome research is a quickly developing field in biomedical research, and we have witnessed its potential in understanding the physiology, metabolism and immunology, its critical role in understanding the health and disease of the host, and its vast capacity in disease prediction, intervention and treatment. However, many of the fundamental questions still need to be addressed, including the shaping forces of microbial diversity between individuals and across time. Microbiome research falls into the classical nature vs. nurture scenario, such that host genetics shape part of the microbiome, while environmental influences change the original course of microbiome development. In this review, we focus on the nature, i.e., the genetic part of the equation, and summarize the recent efforts in understanding which parts of the genome, especially the human and mouse genome, play important roles in determining the composition and functions of microbial communities, primarily in the gut but also on the skin. We aim to present an overview of different approaches in studying the intricate relationships between host genetic variations and microbes, its underlying philosophy and methodology, and we aim to highlight a few key discoveries along this exploration, as well as current pitfalls. More evidence and results will surely appear in upcoming studies, and the accumulating knowledge will lead to a deeper understanding of what we could finally term a “hologenome”, that is, the organized, closely interacting genome of the host and the microbiome.

## INTRODUCTION

With between three- and ten-fold bacteria colonizing our own body (Sender et al., 2016), most of which are in the gastrointestinal (GI) tract (Qin et al., 2010; Zhu et al., 2010), it is hard to imagine that our genome does not devote a particular set of genes to dealing with all the potential threats, as well as coordinating benefits with our microbiome. Indeed, there are many indications of gene-microbiome cross-talk in humans, other animals (Kurilshikov et al., 2017) and even plants (Lundberg et al., 2012), with a large majority of those identified before the wide application of next-generation sequencing. Those genes function in the immune system (Hooper et al., 2012), with good reason: pathogens, an important part being bacteria, were one of the largest forces shaping the evolution of human genomes and thus the survival of our species and other species that rely on the immune system to defend against those pathogens (Kau et al., 2011).

In natural populations of animals and plants, the occurrence of epidemics constantly wipes out populations at the local (leading to disappearance of a species within an area) or global scale (leading to extinction). However, once there are survivors in those epidemics, there are usually genetic explanations in their genomes, such as natural variations in immune-related genes that lead to the higher resistance and survival of a particular group of individuals (Brinkworth and Pechenkina, 2013). In the next generations, those alleles (one variety of a gene) would usually increase in frequency and lead to changes in population genetics (Prugnolle et al., 2005). There are a lot more pathogens that are not as dramatic as those involved in epidemics but that lead to less lethal infections and only the lower fitness of a few; however, these pathogens can still contribute to the change in allele frequencies (Barreiro and Quintana-Murci, 2010). Of course, pathogens are also involved in the arms race of the host and pathogens; no allele can be the perfect solution, but instead, different alleles are selected and enriched in different periods (Novembre and Han, 2012).

**Figure 1:**
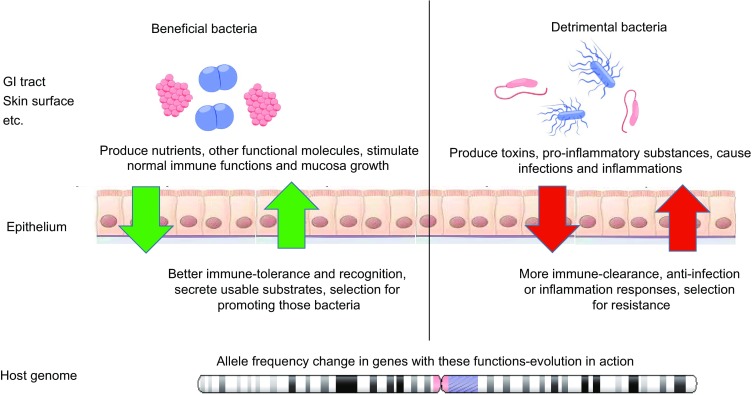
**A simplified illustration of the host gene-microbiome interactions at the interface of various types of epithelia**. The mucosal layer of the GI tract, airway, skin surface and reproductive tract surface are the primary interfaces of host-microbe interactions. Those microbes that we consider as beneficial usually produce nutrients, essential functional molecules and maintain the normal functions of the immune systems; thus the primary aim of host genes is to ensure their immune tolerance and facilitate their growth by secreting mucus, etc. as substrates. While harmful bacteria usually produce toxins, pro-inflammatory molecules and lead to infections, the host genes must clear them from the normal community and defend against inflammation and infections

In humans, we know particularly well what the biggest threats were in our past and now, because of historical and medical records, and we see them in our genomes (Barreiro et al., 2008). Bubonic plague used to decimate one-third of European populations at a time, and its effects are visible in the current populations of Europeans, including some unexpected consequences that are summarized in the book the “*Survival of the Sickest*” (Moalem and Prince, 2008). This is still happening, although on a smaller scale nearly every year. At present, tuberculosis (TB) infects millions throughout the world, mainly in undeveloped areas (World Health Organization, 2016); these infections were more widespread in the past, before the invention of antibiotics. A study by Jostins et al. (2012) found surprising results demonstrating that the genes we think are causal in inflammatory bowel disease (IBD) (mainly composed of Crohn’s disease and ulcerative colitis, and auto-immunity, which affect a small percentage of the population in Europe) turned out to be consequences of selection by TB. Those genes either promote our immune systems’ attempt against TB by lowering the sensitivity to infection or blocking the recognition sites by bacteria, via as-yet-unknown mechanisms; these were consequently under selection by pathogens and are changing in frequency in the population (Jostins *et al.* 2012). Cholera, bacterial and meningitis are among the hundreds of recurring bacterial infections we are aware of, many of which have left a mark in our genome (Gupta, 2016) (Fig. 1).

However, it is not always about bad bacteria. Especially in the last decade, we have started to understand the composition and functions of complex microbial communities in the GI tract of humans and animals (Spor et al., 2011), as well as the skin (Grice and Segre, 2011) and oral microbiome (Dewhirst et al., 2010), reproductive (Ravel et al., 2011) and respiratory systems (Dickson and Huffnagle, 2015). Additionally, we have begun to appreciate the important functions of the normal microbiome in our own health (Fig. 1). We rely on our gut microbiome for digesting food and metabolizing large molecules to smaller ones, so our intestines can take them up more easily (Kau et al., 2011). They produce a large amount of other substances, including vitamins, serotonins, and many other functional molecules that modulate various systems in the host body (Kau et al., 2011; Kostic et al., 2014); thus, the concept of the gut-brain-axis (Foster and Neufeld, 2014), gut-liver-axis (Ray, 2017) and gut-lung-axis (Budden et al., 2017) have been proposed, examined and accepted by wider audiences. The microbiome stimulates the early maturation of the immune systems in infants while maintaining the normal immune functions of adults; meanwhile, many of the immune-related diseases are primarily caused by dysbiosis in the microbiome (Kamada et al., 2013). The hologenome concept, endorsed by many in this field, can be understood to be the comprehensive inclusion of this whole interaction, cooperation and mutual selection at the genomic and metagenomic level, where the host and its microbiome compose a functional entity and the basis for natural selection and evolution (Zilber-Rosenberg and Rosenberg, 2008).

## INDIRECT EVIDENCE

We aim to take the readers along the historical path of discovering the gene-microbiome cross-talk, although the studies we include here are not strictly chronological. For instance, we already knew a number of genes that were critical in maintaining host defence against pathogens (Major Histocompatibility Complex, MHC) (Neefjes et al., 2011), sensing microbes (Toll-Like Receptors, *TLR* for instance, which senses a wide range of molecules produced by microbes) (Kieser and Kagan, 2017), or were involved in other important process that could lead to disease. However, these are largely based on natural knock-out models, i.e., a mutation that leads to loss-of-function of a particular gene. We have studied mice or humans that are usually unhealthy, because critical genes in the host-microbe cross-talk are no longer functional and thus represent the extremities of the gene function spectrum. The more general observations of how variations in the whole genome, especially neutral or near-neutral alleles (those who do not carry as deleterious effect as the loss-of-function) and their association with effect on the microbiome have only come relatively recently (e.g., Hov et al., 2015, Wang et al., 2016, Bonder et al., 2016, Turpin et al., 2016, Goodrich et al., 2016).

The Ochman group published in *Plos Biology* a study on hominoids—primates, including humans, showing that microbiome divergences are well aligned (congruent) with the phylogeny of the mitochondria genome, a relatively simple yet powerful sub-genome for host phylogeny (Ochman et al., 2010). This work was followed by several other in-depth studies (Moeller et al., 2014; Nishida and Ochman, 2017). The microbiome divergence in this study was approximated with the Unifrac distance (Lozupone et al., 2011), which is also a phylogenetic measure of overall bacterial relationships, taking into account both the abundance of bacterial taxa, as well as their positions in a phylogenetic tree. Then, the overall microbiome differences are also clustered to form a “phylogeny” showing their relative similarities, and the congruence with the host phylogeny indicates that the microbiome differences could indeed be shaped by host genetic differences. However, it has to be noted that the evidence here is not without potential confounders, especially considering the natural drift of the microbiome together with its host during evolution and divergence, as well as the dietary differences of different host species, geographical isolation, etc. (Bodenstein & Theis, 2015). This has also been noted in other comparative studies that try to distinguish signatures of genetic affinity from environmental similarities, especially diet (Ley et al., 2008). Nonetheless, it opens the door to understanding that in the whole genome, variations in the hosts underlie gut microbial variations, at least between different species.

The Ley group carried out another landmark study using the UK Twins biobank (Goodrich et al., 2014). The general idea is quite straightforward: by contrasting twins that are genetically identical (monozygotic twins) or non-identical, but related (dizygotic twins), one can quickly determine if some traits, in this case the microbiome, are genetically related. The assumption is that environmental differences are minimal in twins, or at least not extremely different between monozygotic twins and dizygotic twins. When a trait is more similar in the former than the latter, it must be due to genetic similarities. They indeed found this in the human gut microbiome in UK Twins, through a series of consequent studies using 16S rRNA and metagenomic analysis (Goodrich et al., 2016; Xie et al., 2016). A few particular bacteria also showed considerably high heritability, defined as the similarity of a trait due to the same genetic make-up, including one group, Christenalleaceae, that is inversely correlated to body-mass-index (BMI). Mouse models indeed show that this group of bacteria has an effect of reducing obesity. However, it is rather disappointing that further analysis locating the genetic loci corresponding to this group of bacteria did not result in a definitive gene, which could be due to the small sample size of twins. This is because genome-wide-association studies (GWAS), as we are going to describe in detail, usually require relatively unrelated individuals, and in twins, the effective sample size is halved and would not reach one thousand. Org et al. (2015) performed similar analysis in 113 strains of different mice, where the microbiome is also more similar within the same strain than between different strains. They estimated the heritability of the microbiome, taking into account the relatedness of those mice strains as well as the pedigree, and concluded that host genetic variation can explain a substantial amount of variation in the gut microbiota.

## DIRECT EVIDENCE: DESIGNED GENETIC STUDIES

### Resolving confounders

Contrary to the genetic makeup of the hosts, which are (relatively speaking) stable, the microbiome tends to be a dynamic system that has its own natural fluctuations and is highly affected by a variety of environmental factors (Hall et al., 2017); thus, the microbiome observed at different time points within a particular individual could be vastly different. Additionally, when looking at cross-sectional studies, as most of the large-scale investigations are due to the limitations set by studies of such scale, we are examining a snapshot of the microbiome within different individuals, with a high degree of randomness and noise (Walter and Ley, 2011). However, this is similar to a lot of fields in biology: we depend on biological signals that are substantial enough to be picked up by the appropriate detection methods, which, in this framework, includes statistical methods. Additionally, we depend on a sufficiently large sample size to distinguish statistically significant signals from the rest.

Nonetheless, accounting for the most important confounders is essential for any genetic study, for not doing so would lead to type I errors (false positives, where false genetic loci show up as significant) and type II errors (false negatives, where real genetic loci are covered by noise). In mouse models, we could control those to minimize them, while in humans, this would require a systematic investigation of confounders. A large collection of studies has reported anthropometric measures, including age (Yatsunenko et al., 2012), body mass index (BMI, Dominianni et al., 2015), waist measures and dietary habits (David et al., 2014; Dominianni et al., 2015), other life habits and so on (Yassour et al., 2016). In 2016, two studies appeared simultaneously in a special issue in *Science*, in Belgian Flanders (Flemish Gut Flora Project) (Falony et al., 2016) and Northern Netherlands (LifeLines-DEEP cohort) (Zhernakova et al., 2016), in which scientists carried out population-based analyses of confounding factors in shaping the diversity of the microbiome. In this study, hundreds of different measures were tested, filtered and ranked with their respective contributions to the overall dissimilarity of the gut microbiome (beta-diversity) and taxa abundances—the collective property of which is called alpha-diversity—as well as functional capacities. Many of the factors investigated were partially genetically determined, including gender, BMI, blood chemistry, etc., and thus already indicated a genetic involvement in shaping the microbiome. Other factors, such as age, are certainly not genetically determined, but are some of the top contributors and must be accounted for in studying genetics.

Now it might sound odd, that we would also need to control for genetic confounders while studying genetics. The rationales are as follows: in quantitative genetic studies using either crosses (quantitative trait loci, QTL) or natural populations (genome-wide-associations studies, GWAS), we are aware of the fact that the similarity of a trait could be due to overall relatedness. For instance, mice from the same breeding pair share largely the same growth environment and could have a shared microbiome from maternal transmissions (Benson et al., 2010; Wang et al., 2015). Related human individuals might also share a similar microbiome for the same reasons (Goodrich et al., 2016). Conversely, if the populations we study are not well-mixed, but subpopulations exist, thus providing a distinct population structure, the traits we find to be different between individuals might not be due to the effects of a few genes but rather longer term history of evolution, separation, drifts and so on (Yatsunenko et al., 2012). It is essential to account for kinship in QTL studies and GWAS analysis, and to thoroughly determine if there is distinctive population structure. Usually, all but one related individual are removed in a GWAS, and many try to keep the studied population as homogeneous as possible; however, there are also mathematical solutions that take kinship into account, or population structure via the genetic principle components (Kang et al., 2008; Price et al., 2010).

We quickly discuss the methods to account for confounders but will not go into much technical detail. When we investigate univariate traits, such as richness or taxa abundances, for most of the significant confounders, we use linear model/generalized linear models to remove their “effect” and keep the residues for the genetic analysis. This is relatively straightforward but sometimes cannot be well thought through, as many microbiome responses to a factor are non-linear (Lahti et al., 2014); however, other non-parametric factor do not necessarily perform better and can be misleading in its residues. For overall microbiome dissimilarities or beta-diversities, one can also remove the confounding effects of particular factors using constrained principle coordinates analysis (PcoA) and take its residue (also a distance matrix) (Ruhlemann et al., 2017). We rarely see it being performed, mainly because only a few have worked on the beta-diversity association with the host genome to date, and the field is still in its infancy.

### Candidate gene approach

For historical, medical and political reasons, IBD continues to be the central focus of many microbiome investigations. It is a prevalent chronic inflammatory disorder of the GI tract in Europe, with occurrence approximately 1% and is particularly high in certain population of Jewish decedents (Hanauer, 2006). A continuous line of genetic studies have revealed a long list of potential genetic risk factors in IBD patients, including *NOD2*, *CARD9*, *ATG16L1*, *IRGM* and *FUT2*, among others (Xavier and Podolsky, 2007). Since there is a high proportion of microbiome factors in IBD disease, many of those risk genes have been tested to determine if they have impact on the microbiome (Kostic et al., 2014). Many IBD genetic risk factors are indeed are significantly associated with the decrease in the genus *Roseburia*, which plays an essential role in the conversion of acetate-to-butyrate compared to healthy controls, and this genus is known to be decreased in IBD patients (Morgan et al., 2012). We have summarized genes that were hypothesized to have impacts on the microbiome and were consequently tested in either humans (natural genetic variations) or mice (knockout models). As we can see, most studies are still focused on IBD. Of course, this list is by no means complete but contains the most prominent examples we are aware of (Table 1).

**Table 1:** **Examples of candidate-gene approach studies in host gene-microbe interactions**. We performed a literature search centred around the host gene, microbiome and diseases and have listed the most prominent examples where hypothesis-driven studies were carried either in humans (using natural variations) or mice (knock-out models) with respect to changes in the microbiome. We listed the changes observed, as well as the study context (type of disease), which we can see the primary focus on IBD

Gene name	Traits associated with genetic variations	Context of study	References
Human			
*IL13*/*CD14*	Interaction with cesarean delivery and prenatal exposure to antibiotics to affect skin microbiome	Atopic dermatitis	Lee et al. (2014)
*FUT2*	Airway microbiome (*Pseudomonas aeruginosa*)	Bronchiectasis	Taylor et al. (2017)
*IL6*	*Helicobacter pylori*	Dyslipidemia	Pohjanen et al. (2016)
*ATG16L1*	*Fusobacteriaceae*, *Bacteroidaceae*, *Lachnospiraceae*, *Enterobacteriaceae*, *E*. *coli*	IBD	Sadaghian Sadabad et al. (2015)
*CARD9*	Gut microbiome composition	IBD	Lamas et al. (2016)
*FUT2*	Gut microbiome composition, diversity and structure	IBD	Rausch et al. (2011a, b)
*NLRP12*	Gut microbiome diversity	IBD	Chen et al. (2017a, b)
*NOD2*	Gut microbiome composition	IBD	de Bruyn et al. (2017)
*SLC39A8*	Gut microbiome composition	IBD	Li et al. (2016)
*TNFSF15*	Prevotella	IBD	Nakagome et al. (2017)
*SI*	Blautia, Oscillibacter, Ruminococcus and unclassified *Enterobacteriaceae*	IBS	Thingholm et al. (2018)
*IFN-I*	Microbials related to tryptophan metabolism	Multiple sclerosis	Rothhammer et al. (2016)
*DEFB-CN*	Nasopharyngeal bacterial colonization patterns	Otitis media	Jones et al. (2014)
*A2ML1*	Middle ear microbiome	Otitis media	Santos-Cortez et al. (2016)
*C4B*	Gut microbiome composition	Paediatric inflammatory bowel disease	Nissilä et al. (2017)
*CARD15*	Periodontal microbiota in Crohn’s patients	Periodontitis	Stein et al. (2010)
*ELANE*	Subgingival microbiota	Periodontitis	Ye et al. (2011)
Mouse			
*Myd88*	Diversity, segmented filamentous bacteria	Anti-microbial signalling	Larsson et al. (2012)
*Vdr*	*Lactobacillus*, *Clostridium*, *Bacteroides*, *Alistipes*, *Odoribacter*, *Eggerthella*	Bile acid metabolism	Jin et al. (2015)
*Tnf*	Gut microbiome composition	Colitis	Kozik et al. (2017)
*Can*	*E*. *coli*	Colorectal cancer	Peuker et al. (2016)
*Lcn2*	*Alistipes*	Colorectal cancer	Moschen et al. (2016)
*Ifnar1*	Gut microbiome composition	IBD	Tschurtschenthaler et al. (2014)
*Il10*/*Tlr4*	Gut microbiome composition	IBD	Ward et al. (2016)
*Il2*	*E*. *coli Nissle, B*. *vulgatus* and *E*. *coli mpk*/*B*. *vulgatus*	IBD	Bohn et al. (2006)
*Nlrp12*	Gut microbiome composition	IBD	Chen et al. (2017a, b)
*Sirt1*	Gut microbiome composition	IBD, colorectal cancer	Lo Sasso et al. (2014)
*Muc2*	Gut microbiome composition	Ileal homeostasis	Sovran et al. (2015)
*Mhc*	Gut microbiome composition	Immunology	Kubinak et al. (2015a, b)
*B4galnt2*	Gut microbiome composition and Salmonella susceptibility	Inflammation	Rausch et al. (2015)
*TREM-1*	General dysbiosis in gut microbiome	Inflammation	Kökten et al. (2018)
*Nod2*	Gut microbiome under high fat diet	Obesity	Rodriguez-Nunez et al. (2017)
*Fut2*	Multi-generation dynamics of gut microbiome	Susceptibility to enteric infection	Rausch et al. (2017)

Two of the genes are, interestingly, determinants of surface glycans, which serves as the initial contact point/molecule for host-microbe cross-talk. First, the gene *FUT2* encodes an enzyme fucosyltransferase-2 involved in the expression of ABO blood group antigens found on the GI mucosa and secretions. It is found to have two distinct genotypes, one functional secretor and one loss-of-function mutation leading to a non-secretor (McGovern et al., 2010). Recent study revealed that the *FUT2* secretor status (defined by the genotype) has a significant influence on the gut microbiota (Rausch et al., 2011a, b); thus, the genus *Blautia* is lower in group A-secretors compared with non-A-secretors and this reduction is accompanied by higher abundances of members of Rikenellaceae, Peptostreptococcaceae, Clostridiales, and *Turicibacter* (Gampa et al., 2017). Interestingly, the mouse gene B4galnt2 (encoding glycosyltransferase β-1,4-N-acetylgalactosaminyltransferase 2) has similar function in terms of determining the sugar composition of the intestinal mucosa, and it is tissue-specific when we consider the expression patterns. Its expression in the intestine or not is strongly associated with altered bacterial community composition in the mouse model (Staubach et al., 2012). B4galnt2 intestinal expression changes the gut microbiome and consequently facilitates epithelial invasion of *Salmonella typhimurium*, the underlying mechanism of which could be by increased intestinal inflammatory cytokines and infiltrating immune cells. Additionally, B4galnt2 has an interesting pattern of selection in the mouse population that we will discuss towards the end.

Another set of examples are genes responsible for sensing microbes and triggering down-stream cell signalling pathways. Those are usually components of the innate immune system. For example, exogenous microorganisms can be recognized by pattern recognition receptors (PRR), including but not limited to Toll-like receptors (TLR) and *NOD*-like receptors (Kieser and Kagan, 2017), and the MyD88 protein encoded by the *MYD88* gene as an adaptor can modulate the signal transduction pathway. Those genes all have knockout mouse models, and the impact on the gut microbiome has been observed. In addition, we are aware that MyD88 signalling is critical for the development of type I diabetes (T1D), but the incidence of this disease can be decreased in mice by exposure to microbial stimuli, such as injection with mycobacteria or various microbial products, suggesting that the cross-talk by specific genes is essential for the healthy development of immune systems in the early stages of life (Wen et al., 2008; Kostic et al., 2015).

However, the most intriguing case so far is the MHC loci, wherein humans consistently fail to find significant associations with gut microbial compositions, either in candidate gene approaches or even in the recent GWAS (see later). The largest study so far was carried out in Norway using the bone marrow registry to distinguish candidates of different MHC alleles, and the collected microbiome did not reveal a significant difference (Hov et al., 2015). However, it is another story in mouse models and the signals are much more prominent (Kubinak et al., 2015a, b). This highlights some of the difficulties in studying human genetics in terms of its impact on the microbiome, and the effects of certain genetic variations might be very small (and indeed confirmed in following studies) and may be masked by environmental differences. In mouse models, those factors are better controlled. Additionally, we admit that we do not have the comprehensive list of genes that have been studied using the candidate approach, and we merely touched the classic few (Table 1). However, the principle holds and we do expect to see a larger collection of such studies, each with deeper insights into the mechanisms of gene-microbiome interactions.

### Quantitative genetics

**Figure 2:**
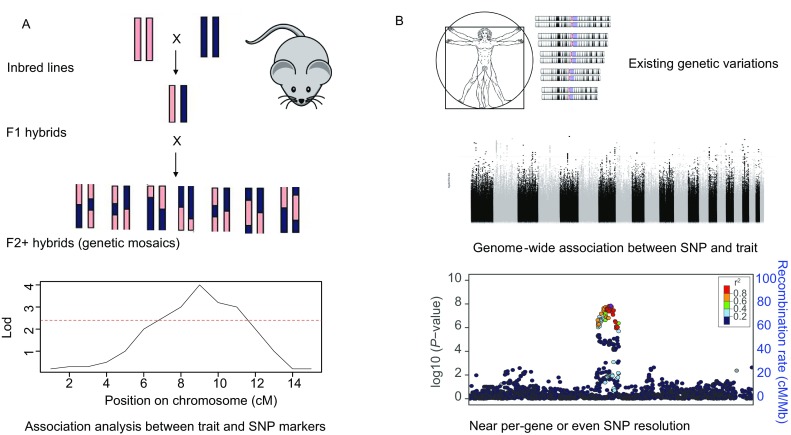
**Schematic overview of quantitative trait loci (QTL, panel A) and genome-wide-association studies (GWAS, panel B)**. Both work on genetic variations, but result from different processes, either by intentional crosses (QTL) or extant (GWAS), and the linkage blocks are of a different size due to the number of recombinations. Association tests were carried out for SNPs and interpolated for a region in QTL analysis, while in GWAS it is done for each SNP and a “peak” in the Manhattan plot indicates a haplotype that might be significantly associated with the trait. In both cases, usually the *P*-values were transformed into −log(*P*) to indicate the significance level, and the genome-wide significance for QTL is usually determined by permutation tests. For GWAS, it is commonly accepted to be set at 1E−08 to 5E−08

The tools of quantitative genetic studies come in handy when we intend to screen the associations between the microbiome and the whole host genome, instead of individual genes. Largely roots from plant and animal breeding science, quantitative genetics aims to find genes or genetic loci that are underlying important biological traits (phenotypes) of studied organisms, providing the basis for causal, mechanistic studies as well as practical applications (improve the production of crops or animals, for example) (McCarthy et al., 2008). Two terms are widely used: QTL and GWAS (Fig. 2). Many argue that, in the strict sense, they are mutually exclusive and that the former applies to quantitative traits such as height in animals or yields in crops, mainly using planned crosses as the study cohort and that resolution is proportional to the generations of recombination, while the latter applies mostly to humans where the natural population history of a thousand generations has led to considerable recombination. However, it does not reach the per gene level. Instead, human genomes still have large blocks of genes that are linked, and little recombination has occurred yet. On the contrary, linkage disequilibrium (LD) also occurs as a result, which can lead to gene-level or even SNP-level resolutions for associations; in many cases, the studied trait is binary, especially about disease. However, we would like to note that mathematically, the two approaches are essentially the same. Quantitative genetic studies are about finding significant associations between genetic variations, either a single SNP or a large chromosome region. Both cases consider LD information, and variations in a defined phenotype and different types of traits only affect the model of the association tests. Binary traits require logistic regression, and the result of the associations are usually denoted as an odds ratio (OR): compared to the basal frequency of a trait, a particular SNP could change the frequency of that trait by OR fold. Thus, it is enriched, if OR is higher than 1, and vice versa. While continuously distributed traits require linear or similar regressions and the “effect” of SNP/haplotype block are beta-values or z-scores, depending on the model used. This means that the mean value associated with a particular SNP/haplotype block deviates from the overall mean, measured by the variance (Hirschhorn and Daly, 2005).

**Figure 3:**
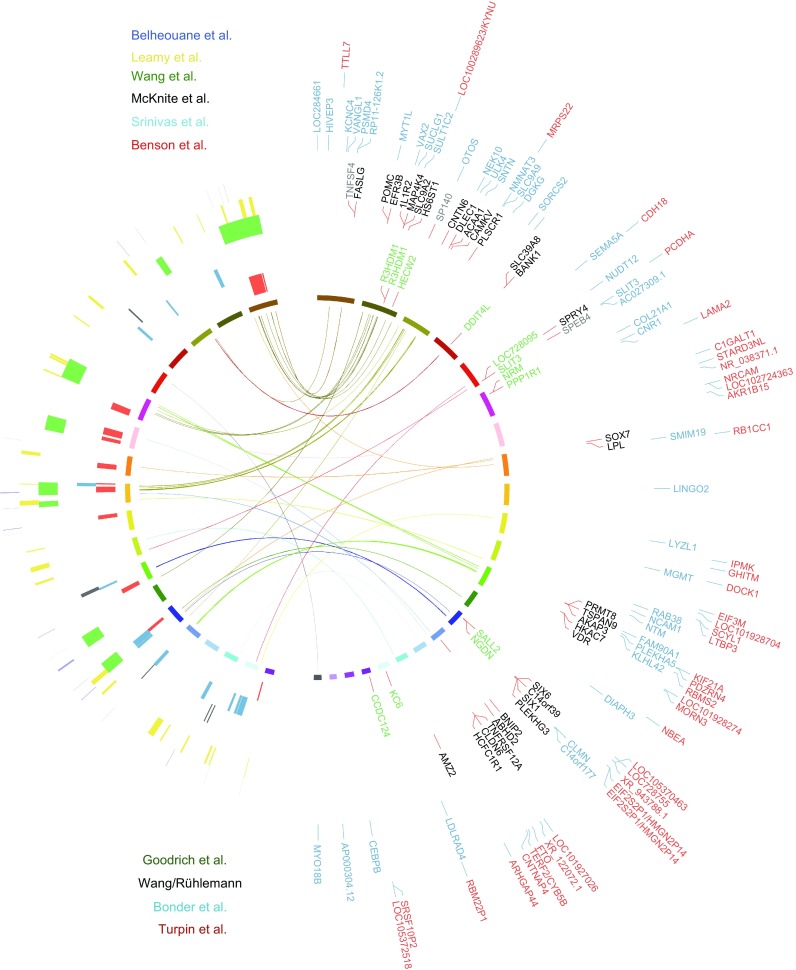
**Overview of microbiome QTL in mouse and GWAS in humans to date**. Left half shows the six QTLs in mice, coloured by different studies and the confidence intervals are marked on the mouse chromosome. Please note that Belheouane and Srinivas studies are skin microbiome QTLs. The right half shows the genes implicated in human GWAS, including UK Twins, FoCus/PopGen (both original publication and later with modified methods), LifeLines-DEEP and GEM studies. Links in the middle show potential overlapping genes that showed up in human GWASs and fall into a confidence interval in mouse QTLs, which might be supportive of each other in terms of the association with microbiome variations

To date, we are aware of six QTLs (Fig. 3), using crosses, that were carried out in mice as the model organism with the microbiome as the targeted trait. Four QTLs were done for the gut microbiome, while the remaining two focused on the skin microbiota. Benson et al. in 2010 published the first proof-of-concept study, showing that in a mouse cross of several generations, we can indeed locate the genetic variations at certain chromosomal regions to the variations of gut microbiome. Even when the resolution is not high, there are some interesting hints about the potential genes involved that correlate to specific microbiome abundances, in particular genes downstream of toll-like-receptor 2 (Tlr2), a gene that is mainly responsible for sensing gram-positive bacteria and downstream genes, including Irak3, Lyz1, Lyz2, IL-22, and IFN-gamma, while the correlated microbiome traits are indeed Coriobacteriaceae and *Lactococcus* (Benson et al., 2010). McKnite et al. (2012) and Leamy et al. (2014) followed up using different cross schemes and identified more regions with limited overlaps between the studies, each with some interesting insights about the genes that might be involved. Wang et al. (2015) published another interesting study using hybrid mice as the QTL cross cohort, where two subspecies of house mice are crossed to the second generation, and many regions are found to be correlated to microbial diversity. Lab mice are essentially *Mus musculus domesticu*s, while its eastern European counterpart is *Mus musculus musculus*. They occur naturally in central Europe and have a well-studied evolution and speciation system. Currently, it seems that the microbiome is also affected by this hybridization. Moreover, the type of association is interesting. Half of the associations are transgressive, meaning that heterozygotes for a particular genetic locus have abnormally high or low values, out of the range for both homozygotes (Wang et al., 2015). This and one potential epistatic interaction that follows the Dobzhansky-Muller incompatibility model reveals further insights that the microbiome shape the genome evolution of hosts. Details can be found in the publications and in additional literature by the Bodenstein group (Brucker and Bordenstein, 2013; Bordenstein and Theis, 2015).

The other two skin microbiome QTLs, both published by the Baines group, provide interesting observations of host-microbiome homeostasis on the surface of the body (Srinivas et al., 2013; Belheouane et al., 2017). Working on an auto-immune skin disease model called epidermolysis bullosa acquisita (EBA), the group has extended the previous disease-oriented QTLs to include microbiome composition and found that the microbiome could indeed play an important role in determining disease manifestation. Even with roughly the same genetic makeup, developing the disease or not is correlated to the abundance of *Staphylococcus*, a potentially protective species. In contrast, when the bacterial abundance is taken into account in the statistical model, the power of QTL significantly increases, as the “noise”, or environmental confounder of bacteria, is controlled (Srinivas et al., 2013). The second skin QTL has innovated the study approach and used 16S rRNA gene transcripts, which examine the “active” part of the microbiome instead of the standing communities. Together with further cross generations (15th instead of 4th), this resulted in an almost per gene resolution and more significant associations when the transcripts are used. Additionally, some of the loci are involved in carcinogenesis of the skin, which are correlated to similar bacteria that could also lead to cancers in the colon (Belheouane et al., 2017).

We need to mention that the study by Org et al. (2015) discussed above actually carried out a GWAS in a similar fashion to that performed in humans, and several important genes were identified in this process that are associated with the microbial taxa. Contrary to QTLs above, they used standing variety of mouse strains (110 of them) instead of crosses that are specially set up, and the methods carefully considered the population structure. The only limitations were the relative small sample size and the low number of SNPs, for which we cannot really reach a similar genome-wide significance threshold in humans (will discuss below). This limits the resolution in the results.

The microbiome-oriented GWAS in humans, coincidentally also have six cases so far. We would consider at least two not to be sufficiently large to be considered equally as the remaining few. The first approximation of a microbiome GWAS was not really by design. Rather, Blekhman extracted human genome reads from HMP metagenomic raw data, called SNPs from those human reads for each subject, and correlated the genetic variations of the hosts to the microbiome variations. One particular association is between the lactase (*LCT*) gene and *Bifidobacterium*, and both are related to milk consumption and thus could understandably be correlated (Blekhman et al., 2015). However, whether the “fished out” human reads were sufficient to carry out proper SNPs was never clear, and neither was the reliability of the consequent analysis. Davenport et al. (2015) reported a small, but more conventionally designed GWAS study and managed to find some associations, none of which reached the commonly accepted genome-wide significance threshold (which is 5E−08 or 1E−08, the rationale is that when you screen millions of SNPs, the real significance should stand Bonferroni or Benjamini-Hochberg corrections for multiple testing, and thus it is commonly set at this scale). The Ley group also continued with their endeavours in the UK Twins cohort with multiple models for microbiome-SNP associations, and they did manage to actually find some hits that were later rediscovered, including *LCT* and *SLIT3*. However, because of the lack of a central focus on the models or functional studies, the study did not go into sufficient detail in exploring gene-microbiome ties at the genome scale (Goodrich et al., 2016) (Fig. 3).

The real breakthrough in human GWAS on the gut microbiome came as a trio in the November issue of *Nature Genetics* in 2016, where a German cohort (PopGen/FoCus) (Wang et al., 2016), a Dutch cohort (LifeLines-DEEP) (Bonder et al., 2016) and a Canadian cohort (GEM) (Turpin et al., 2016) simultaneously published large-scale GWASs on the human gut microbiome (Fig. 3). All three cohorts include more than 1,000 unrelated individuals, all have replication cohorts, and all have considered the distribution properties of bacterial taxa (only a small fraction fits normal distributions, the rest are mainly zero-inflated). Among these three studies, the German and Canadian studies used a two-part hurdle model to address zero-inflations, while the Dutch study worked on the none-zero part when this was the case. The other difference is that the German/Canadian cohorts worked on a 16S rRNA based bacterial composition, while the Dutch study also has shot-gun metagenomic data and thus could map certain functional pathways. Beyond using bacterial abundance as the main studied trait, the German study, in particular, proposed a method to associate the overall microbial diversity (beta-diversity) to human genetic variations, and discovered 42 loci that passed the significance threshold, including one Vitamin D receptor (*VDR*) that was known to be involved in bile-acid sensing and homeostasis. Additionally, in this study, a number of functional studies, including bile acid analysis, metagenomic sequencing, cross-checking with different databases and comparing the human transcriptomes vs bacterial abundances (“coupling”), established the validity of *VDR* as a central part of the human-microbiome cross-talk, mostly via bile-acid metabolism and downstream pathways (Wang et al., 2016). The beta-diversity association method was consequently further developed to be less computationally intensive and more adapted to a higher dimensionality, with some further interesting loci discovered in this process (Ruhlemann et al., 2017). The Dutch study mainly confirmed the previous findings of *LCT*-*Bifidobacterium* associations and showed that environmental influence (in this case, milk intake) also interacts with the genotype of the individuals and shapes the microbial abundance (Bonder et al., 2016). Benson wrote a nice summary on all three of these studies, which was published in the same issue of Nature Genetics (Benson, 2016). Additionally, Kurilshikov and Zhernokova pieced together a wonderful review on this extended topic as well (Kurilshikov et al., 2017).

## CONCLUSIONS

We have described the chronicles of genetic investigations in understanding host-microbe interactions, and the main results of the different approaches. We have seen indirect evidence in comparative studies, but those studies have limitations. We could investigate individual genes of interest and gain insights into their importance but could not generate a complete picture. Additionally, there is a quantitative genetic approach, and there are many things that we need to be cautious of. However, this endeavour is, by no means complete. We are just in the preliminary stages of investigation. Here, we would also note the current limitations of the mentioned studies, as well as our own perspectives into future efforts and directions.

### Limitations

Our review is very focused human and mouse studies while ignoring the larger context of other model or non-model organisms. The reason is because of the great deal of effort put into the former two models and that the studies in humans and mice are considerably more relevant to our own health. We do know that a vast collection of literature exists for plant gene-microbe interactions, and many are textbook models, such as those genes involved in the invasion and colonization of *Agrobacterium*, which involves a complex interplay that would dwarf some of the host-microbe crosstalk in animals (Nester, 2015; Ellis, 2017). Similarly, a plant GWAS on the microbiome has been published for *Arabidopsis thaliana* and revealed a list of genes that may participate in a wider scale of interactions as well (Horton et al., 2014). However, since many genes lack counterparts in animals or at least do not carry out the same function, the value as a reference to other organisms is limited.

We do not have a shortage of host-microbe cross-talk examples in *C*. *elegans*, *Drosophila* and Zebra fish and many other common model organisms used in the lab. Most of these are single pathogens, and the difference observed in consequences are due to the genetic variation of the host. This again falls into the category of candidate gene-based approaches, in which one gene was the primary focus of study, and a glimpse into the greater picture of host-microbe cross-talks in those organisms has been observed. In regard to genome-wide, quantitative genetic studies in the microbiome, there have been two carried out in *Drosophila* (Chaston et al., 2015; Dobson et al., 2015), where the authors have pinpointed the interactions of nutrition and the host, and the microbiota serves as an important intermediator for the effects of nutrition to actually occur. Translated into terms that are widely used in human or mouse studies, the microbiome largely determines the metabolomes of the host and consequently the health status. Moreover, there are also a handful of studies, including one on chicken (Zhao et al., 2013), and we apologize if we missed other studies using different studying organisms. All of these studies make important contributions to the field, and by combining those studies, we generate a grander picture and get closer to solving the full puzzle. To achieve this, both the accumulation of data as well as innovation in methods are required.

Still, association does not mean causation, which is a limitation of association-based studies. Functional validation and establishment of real causation is still the bottleneck of many gene-microbe interactions. Moreover, compared to the limited knowledge we have on the host side, we know little to none about which bacterial genes are carrying out the cross-talk with the host. In pathogens, we study the key virulence factors that are part of the invasion process, or pathogenesis, including various toxins, different types of secretion systems, or genes responsible for producing the key metabolites influencing the hosts. We also know a few molecules that play a central role in being recognized by the hosts, such as cell wall components, lipopolysaccharides (LPS). However, we lack a general picture of which part of the bacterial genomes are responsible for establishing and maintaining the connection with the host and which parts underlie the breakdown of such homeostasis. The authors assume that this varies from bacteria to bacteria of course and that environmental bacteria would need fewer genes for this task, while symbiotic bacteria should devote an essential part of the genome; otherwise, they would not be able to maintain a symbiotic relationship with the hosts. The gut microbiome, skin microbiome and bacteria in other body sites are intermediate in the sense that they are not strictly symbiotic but would still need to invest part of the genome. Some studies have shown that long-term intracellular symbionts in insects have lost a large part of their genome in the evolutionary process and only keep a small fraction of the essential genes (Wernegreen, 2002; Bennett et al., 2014). Whether this occurs in the gut or skin is not known, and the authors would argue that this genome reduction, if it exists, would apply to genes that are more maternally transmitted than those that are usually acquired from the environment.

### Outlook

As we proclaimed in the beginning of this review, pathogens are driving forces in allele frequency changes in host populations, and we usually observe the results of this selection. However, this rarely occurs in real time, and we have not conducted an in-depth examination of the exact parameters of fitness and the costs. However, Vallier et al. (2017) carried out an astonishing study showing that, in natural populations of western house mice (*Mus musculus domesticus*), two alleles of the B4galnt2 gene co-exist as a result of long-term balancing selection, where one allele confers protection against various pathogens and thus could be favoured by pathogen-driven selection. However, it also leads to bleeding in the GI tract and could potentially reduce host fitness (this is similar to the human bleeding disorder called type 1 von Willebrand disease and could also be selected because it has beneficial effects during pathogen infections). Because this balancing selection is rather recent (from geographical distribution pattern combined with population history), the authors built up evolutionary models and estimated the fitness costs of the bleeding allele. It turns out that the currently observed allele frequency, as well as distribution, could only be explained when there is a heterozygote advantage and an advantage for homozygotes with bleeding alleles, and the costs in fitness of bleeding counts half of pathogenic infections. This is not biologically relevant proof, of course, as both fitness costs and infection costs are extremely difficult to quantify. However, it shows how important selection from microbes can be and how tiny microbes shift our genome, even leading to alleles that are otherwise detrimental to humans to maintain in the population. This is not the only case, as many of the underlying genes/alleles of autoimmune disorders and metabolic syndromes are believed to be the result of selection by pathogens in the past and will continue to interact and change our genomes in the future (Nielsen et al., 2007; Novembre and Han, 2012; Milot and Pelletier, 2013).

Our review has so far been focused on individual genes, and we could only limit it to the main proof-of-concept studies. An important concept in understanding the host-microbe cross-talk, similar to in any genetic study, is the gene-environment interactions (G by E), where the genetic background manifests different effects when the environmental context changes. This has been shown to be the case in the *LCT* gene and *Bifidobacterium* (Blekhman et al., 2015; Goodrich et al., 2016), where dairy intake serves the environmental background (Bonder et al., 2016). However, we do not have many other examples, since the content of environmental influences is so vast, and many studies have not managed to include a sufficient amount. At the same time, the sample sizes usually do not permit this kind of analysis either. In addition, there is an urgent need to move beyond single gene associations, since for most of the complex traits, the power of the single gene in explaining microbiome diversities as well as functionalities is limited, and conclusions can only be partial and misleading. Integrating multiple genetic variations with respective weight, which results in polygenic scores as used in many diseases (Dudbridge, 2013), could be applied in microbiome research to explain the underlying genetic architecture for a single taxon or the overall diversity and would yield a more complete overview of host-microbe cross-talk. Another important direction is to move beyond single genes to biological pathways, which participate, and examine the association between microbes and certain cellular processes/signalling pathways. This requires enrichment analysis from a collection of single genes (Ramanan et al., 2012). Overall, this fascinating area of research has just revealed its potential in terms of understanding both fundamental biology, as well as application in medicine and human health, with many aspects that have yet to be examined.
